# Electromagnetic fields in the treatment of chronic lower back pain in patients with degenerative disc disease

**DOI:** 10.4155/fsoa-2015-0019

**Published:** 2016-02-11

**Authors:** Amarjit S Arneja, Alan Kotowich, Doug Staley, Randy Summers, Paramjit S Tappia

**Affiliations:** 1 Rehabilitation Hospital, Internal Medicine, Winnipeg, Manitoba, Canada; 2 BioResonance Technology Inc., Winnipeg, Manitoba, Canada; 3 St. Boniface Hospital Research, Office of Clinical Research, Winnipeg, Manitoba, Canada; 4 National Research Council, Institute for Biodiagnostics, Winnipeg, Manitoba, Canada; 5 St. Boniface Hospital Research, Asper Clinical Research Institute, CR3129-369 Tache Avenue, Winnipeg, Manitoba R2H 2A6, Canada

**Keywords:** alternative and complimentary medicine, biotechnology, pain

## Abstract

**Aim:**

To examine the effects of low-amplitude, low frequency electromagnetic field therapy (EMF) therapy in patients with persistent chronic lower back pain associated with degenerative disc disease.

**Design:**

Double-blind, randomized and placebo controlled.

**Intervention:**

EMF using a medical device resonator; control group underwent same procedures, except the device was turned off.

**Outcome measures:**

Pain reduction and mobility.

**Results:**

Improvements in overall physical health, social functioning and reduction in bodily pain were observed in the EMF group. The pain relief rating scale showed a higher level of pain relief at the target area in the EMF group. An increase in left lateral mobility was seen only in the EMF group.

**Conclusion:**

EMF treatment may be of benefit to patients with chronic nonresponsive lower back pain associated with degenerative disc disease.

## Abstract (lay abstract)

**Lay abstract:** In this preliminary study, we examined the possible value of using a medical device that generates low energy electromagnetic fields for reducing pain and increasing movement in individuals with persistent lower back pain associated with degeneration of the discs of the spine. After five treatment sessions (each lasting 1 h) over a 2-week period, it was found that as compared with those individuals that were not exposed, low energy electromagnetic fields at the pain area may not only improve physical functioning, but also may reduce pain.

**Figure 1.**
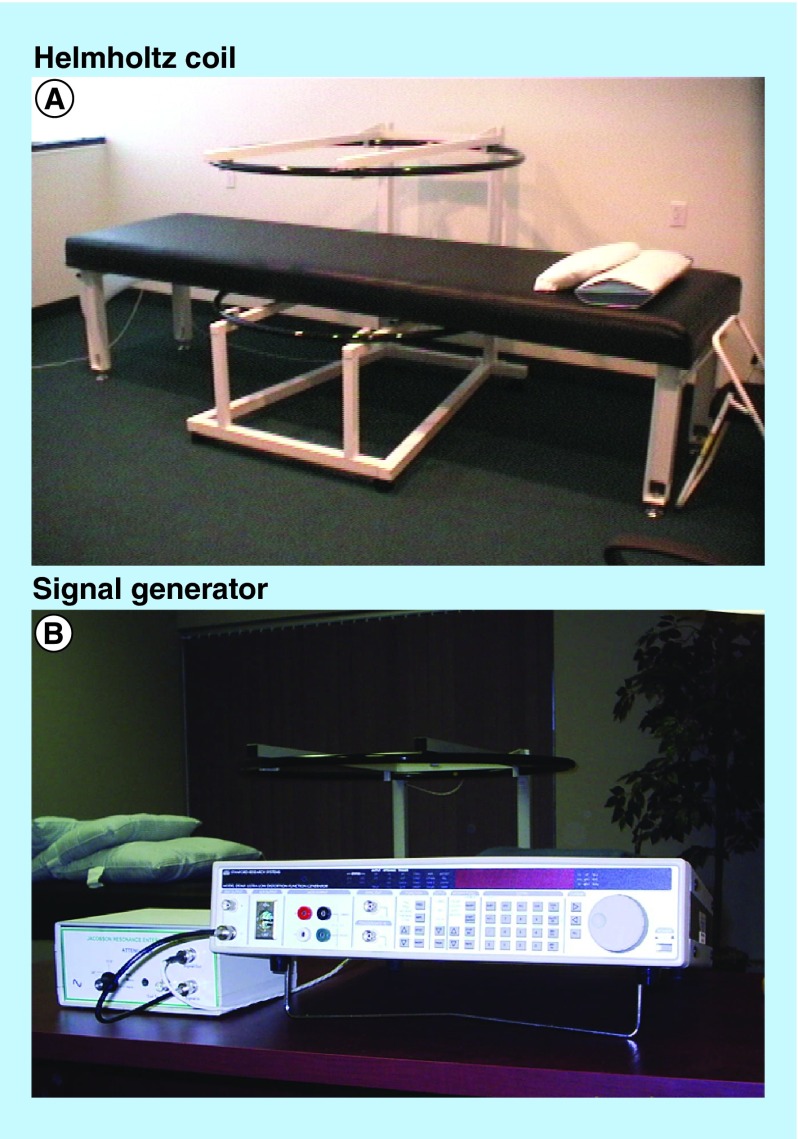
**Medical Device Resonator, showing (A) the Helmholtz coil and (B) signal generator.** The Medical Device Resonator has Health Canada License and CE Mark (#2001010303CPON0318) and was developed using defined physical principles for electromagnetic field therapy production.

**Figure 2.**
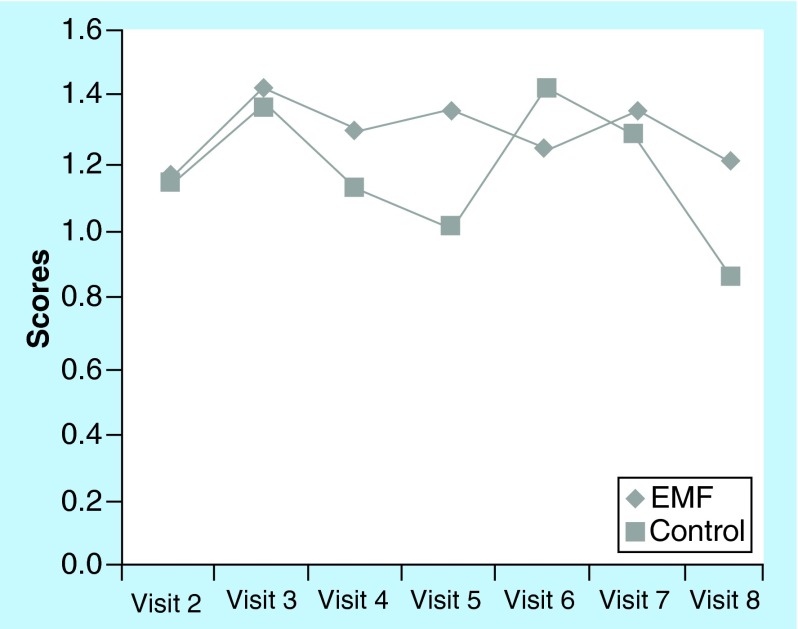
**Pain relief rating scale scores for visit 2 through to visit 8 for control and electromagnetic field groups.** Scores on the pain relief rating scale for visits 2–8 were graphed in order to represent the pattern across time and between groups. Higher scores = greater pain relief. EMF: Electromagnetic field therapy.

**Figure 3.**
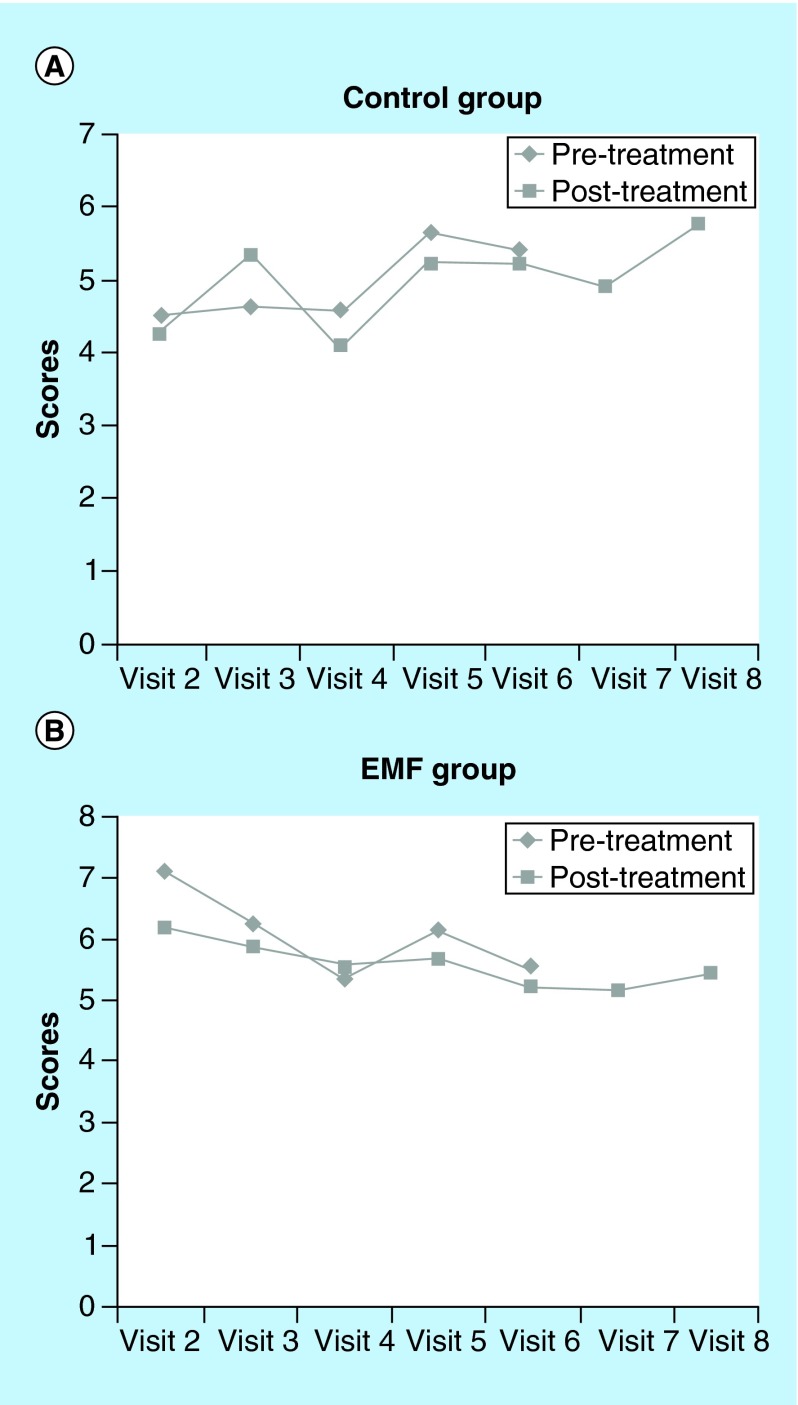
**McGill Visual Analogue Pain Scale pain scores in (A) the control and (B) electromagnetic field groups prepost treatment.** Scores on the McGill Visual Analogue Pain Scale for visits 2–8 were graphed in order to represent the pattern across time and between groups. A plot of all pretreatment pain measures during the study period was constructed. A similar plot for the post-treatment pain measures was also obtained. Higher scores = higher level of pain. EMF: Electromagnetic field therapy.

**Figure 4.**
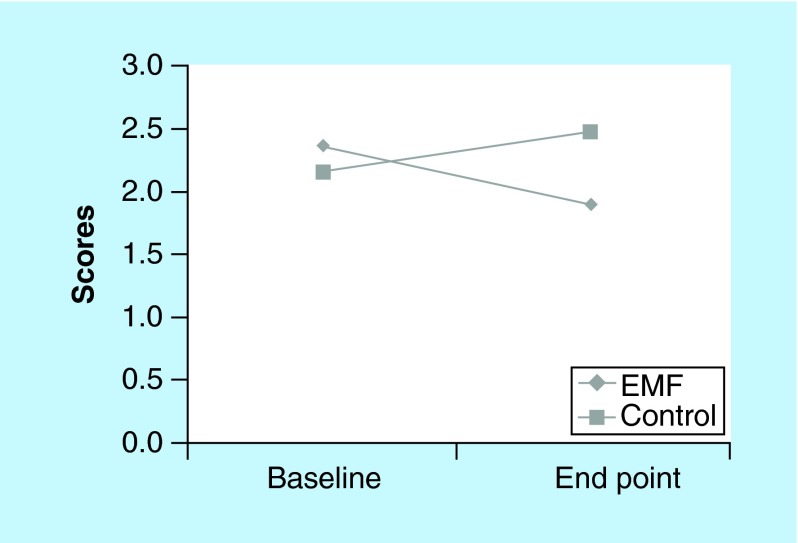
**McGill PPI scores for electromagnetic field and control groups at baseline and end point.** Present pain intensity scores depicted are from baseline (visit 2 pretreatment) to end point (visit 8 post-treatment). Lower scores = greater decrease in pain intensity. EMF: Electromagnetic field therapy.

**Figure 5.**
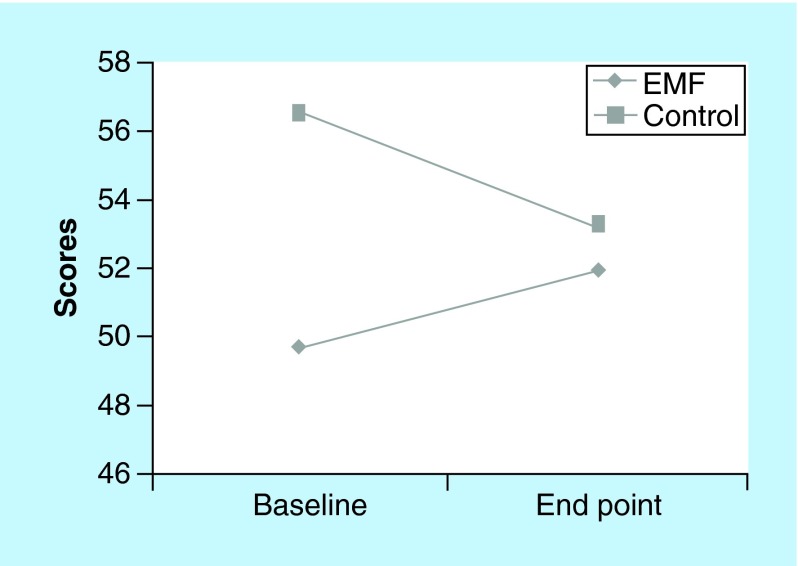
**Left lateral mobility for electromagnetic field and control groups at baseline and end point.** Mobility scores depicted are from baseline (visit 2 pretreatment) to end point (visit 8 post-treatment). Higher scores = greater mobility. EMF: Electromagnetic field therapy.

It has been estimated that up to 84% of adults have low back pain at some point during their lives [1,2]. Intervertebral disc degeneration is a disease of the discs connecting adjoining vertebrae in which structural damage leads to degeneration of the disc and surrounding area [3]. The degeneration of the disc is considered to be a normal process of aging, but can accelerate or be precipitated by other factors [3]. Degenerative disc disease is a strong etiologic risk factor of chronic low back pain (LBP) [4]. Although a number of pharmacological treatment options are available for pain management, the occurrence of side effects, tolerance, noncompliance and contraindications have raised concerns over their use in some patients with LBP due to degenerative disc disease [5]. On the other hand, a number of nonpharmacological therapies for LBP have been employed [6]. In this regard, therapies with evidence of moderate efficacy for chronic or subacute low back pain were suggested to be cognitive-behavioral therapy, exercise, spinal manipulation and interdisciplinary rehabilitation [6]. While for acute low back pain, the only therapy with evidence of efficacy was suggested to be superficial heat [6]. However, electromagnetic field therapy (EMF) has now also emerged as an alternative, safe and effective treatment option for chronic pain in different clinical settings [7–10].

Presently, there is a paucity of information available in the literature that describes the clinical utility of very low EMF in the treatment of chronic LBP associated with degenerative disc disease. Accordingly, in this pilot study, a placebo-controlled, randomized, double-blind study design was employed to assess the efficacy and safety of low-amplitude/low-frequency EMF in the management of nonresponsive chronic LBP and functional capacity in patients with some degenerative disc disease. In addition, quality of life scores as well as mobility measurements were collected to further assess effectiveness of EMF in this cohort of subjects.

## Patients & methods

### Subject recruitment

Twenty one subjects were referred to participate in the study through family physicians, physical medicine and rehabilitation, sports medicine, orthopedics and rheumatology departments from the Health Sciences Centre, Winnipeg, Manitoba. The protocol was approved by the institutional review committee. Subjects were male and female, over 25 years of age who had documented chronic LBP persisting more than 6 months, which was not responsive to conservative therapy, MRI radiographic confirmation of diagnosis (minimum 25% and maximum 75% degeneration, and loss of some disc height) and no herniation. In these subjects, the disc degeneration resulted in discogenic low back pain (due to inflammation and abnormal micromotion instability [11]. In order to minimize confounds and to allow for a stringent study design, several exclusion criteria were applied. Exclusion criteria did not allow for the enrollment of patients with bone-on-bone cases, subjects taking steroids, weighing more than 210 lbs, subjects with pacemakers or with metallic prostheses, pregnancies or with epilepsy, diabetes, uncontrolled high blood pressure, history of congestive heart failure and/or cardiac arrhythmias, stroke, heart valve defects, thyroid conditions, subjects at risk of transient ischemic attacks, blood clots or aneurysms, subjects with past history of major head trauma or brain surgery and subjects with chronic low back pain due to sciatica.

### Subject randomization

Patients were randomly assigned to either the control (n = 7) or EMF treatment (n = 14) arm. Randomization was carried out by the subject drawing an envelope containing a preassigned number. The envelope was handed unopened to the operator of the device. The operator of the device opened the envelope in the control room where no other persons involved in the study were present. Inserts of envelopes containing even numbers were assigned to the treatment arm, whereas inserts of envelopes containing odd numbers were assigned to the control arm. Since the device operates in total silence and there are no other indicators of when the device is on or off, the study was blinded for the patient, and the clinical assistant doing the positioning of the patient. Subsequent visits to the center remained blind as only the operator of the device had the record of what the previously assigned subject number was and which arm of the study the subject was in.

### Intervention & study design

The magnetic fields to be used in this pilot study were generated by a medical device resonator. This resonator is a noninvasive device that does not utilize ionizing radiation and produces a uniform low-amplitude/low-frequency EMF through a Helmholtz coil (Figure 1A) operated through a signal generator (Figure 1B). The coil was positioned over the treatment area of the individual lying in horizontal position. This device has a Health Canada license and is listed as an approved device. Subjects assigned to the treatment group were exposed to EMF for 1 h according to the values defined in the Pattern Protocol (Table 1). Subjects in the treatment group were exposed to five treatment sessions (visit 2 through to visit 6) during a 2-week period, for 60 min per treatment, whereas subjects assigned to the placebo group, underwent all procedures, as the treatment arm, except that the device remained off during the session. We selected a room that was not close to items that generate significant environmental fields, such elevators, heating, ventilation and air conditioning (HVAC) systems, and electrical panels. Each subject rated their pain level before and after each treatment, 1 week and 1 month after the last treatment. Accordingly, post-treatment evaluation was conducted at visit 7 and visit 8 at the 5th and 9th week of the study period. Participants underwent a demographic questionnaire, medical history, the Roland Disability Questionnaire and the SF-36 Health Survey at baseline (visit 1, week 1). The Roland Disability Questionnaire and the SF-36 Health Survey were repeated at the end of the study following visit 8. On visits 2 through 8, participants completed the Pain Relief Rating Scale immediately following treatment. Participants completed the McGill Visual Analogue Pain Scale (VAS) and Present Pain Intensity Index before and after treatment on visits 2 through 8. It is pointed out that on visits 7 and 8 the McGill Pain Scales were only measured post-treatment. In addition, measurements of forward mobility (measured from waist to point on mid-upper back), right lateral mobility and left lateral mobility (side to side measure to floor) were obtained before and after treatment on visits 2 through 8. Pain scores were collected through a manual assessment by the subjects by placing a mark on a continuous line between 0 to 10, where 0 = no pain and 10 = worst pain.

### Statistical analysis

Demographic and medical variables at baseline were compared for differences between the two groups using the independent group *t*-test. Differences in scores on the Roland Disability Questionnaire and SF-36 Health Survey between baseline and end point for each group were analyzed by the Wilcoxon matched pairs test based on ranks. The level of statistical significance was set at p < 0.05. Scores on the Pain Relief Scale and McGill Visual Analogue Scale for visits 2–8 were graphed in order to represent the pattern across time and between groups, as there were insufficient numbers of subjects and not enough statistical power to allow for a more formal statistical analysis such as two-way repeated measures ANOVA. Scores on the McGill Present Pain Intensity Index and forward, left and right lateral mobility were graphed at baseline and end point for the two groups. Differences in scores between groups and across time presented in the graphs are linearly scaled. The data were analyzed by SPSS (version 14.0; SPSS Inc, Chicago, Ill).

## Results

### Patient characteristics, RDQ & SF-36 Health Survey

Demographic and medical variable comparison of EMF and control groups is shown in Table 2. It can be seen that there were no differences in age, blood pressure or heart rate between the two groups. The Roland Disability Questionnaire revealed that the EMF and Control groups had similar Total Scores with nonsignificant differences (Table 3). The SF-36 Health Survey (Table 4) showed a significant improvement in bodily pain (p = 0.045) in the EMF group from baseline to end point.

### Pain relief rating scale, McGill VAS & present pain intensity index

The EMF group reported a higher level of ‘pain relief at the target area’ following treatment as compared with the control group on six of the seven visits (Figure 2). Although pain relief scores were similar for the two groups on visit 2, at end point (visit 8) pain relief scores were much higher in the EMF group. The McGill visual analog pain scale measures “the amount of pain recently experienced in the last 48 h.” The EMF group reported a much higher level of pain in the last 48 h (7.1 vs 4.5 on a 10-point scale) than the control group at the initial visit 2 (Figure 3). While the pain scores for the control group increased from the initial value at visit 2 to the final measure at visit 8 (Figure 3A) the pain scores for the EMF group showed the opposite pattern as pain scores decreased from visit 2 to visit 8 (Figure 3B). Indeed, further observation of the data presented in Figure 3 shows not only the progression of changes in the pain scores at each of the study visits, but is also suggestive that the pain relief may persist between visits as well as in the post-treatment period. This is borne out from comparing post-treatment values of the pain scores with the subsequent visit pain scores and comparing the pain score values for both of the post-treatment visits (visits 7 and 8) to the pain score value at the end of the treatment period (visit 6). Furthermore, while the EMF group showed a decrease in pain intensity from baseline to end point, the control group experienced an increase in pain intensity, as measured by the McGill present pain index, from baseline to end point (Figure 4).

### Forward & lateral mobility

Figure 5 shows the left lateral mobility for control and EMF groups at baseline and end point. At baseline, the EMF group had less left lateral mobility than the control group, however; the EMF group evidenced an increase in left lateral mobility from baseline to the final end point of the study. Both groups showed little change in forward and right lateral mobility following treatment from visit 2 to visit 8 (data not shown).

## Discussion

Several recent commentaries have suggested that EMF treatment may provide moderate benefit in terms of pain relief and/or physical functioning in different pathophysiological conditions [12–16], but no evidence has previously been provided for the use of EMF for treating chronic LBP associated with some disc degenerative disease. Although the sample size of the present study was relatively small, the preliminary results of this pilot study suggest that low energy EMF delivered by a prototype device (Medical Device Resonator) over a 2-week, five treatment session protocol, has some effect in patients with chronic LBP and physical functioning. Specifically, measures of pain relief at the target area, present pain intensity and pain in the last 48 h in subjects with chronic LBP associated with disc degenerative disease, suggest that EMF treatment may be effective in pain management. Furthermore, from a clinical standpoint, it is apparent that there is a reduction in pain scores in the EMG group of approximately 23% (pretreatment, visit 2 value vs post-treatment, visit 8 value), while an increase in the pain score of approximately 31% (pretreatment, visit 2 value vs post-treatment, visit 8 value) (Figure 3), therefore this difference may be of clinical significance. In this regard, while some authors have suggested a 50% change in VAS/NRS as clinically meaningful changes [17], others suggest a reduction of approximately 30% as clinically significant [18].

The present study also provided suggestive evidence that left lateral mobility increased following EMF treatment compared with the controls. Importantly, in the present study, there were no adverse events or side effects related to the device, treatment or protocol reported. An unexpected finding in our study was that despite a significant reduction in bodily pain, physical functioning was reported to be worse in the EMF group when comparing baseline to end point values. This could be explained on the basis that our study was conducted with a small number of subjects and bias in the questionnaire responses may have occurred, thus impacting on the statistical analysis of the data on physical functioning. Furthermore, it is conceivable that questions asked, with regards to certain daily activities, could have been indicated as difficult to perform even with less back pain. Therefore, a larger study is warranted in order to further validate the findings of the present pilot study and control for accidental biases.

Although EMF in the 150 Gauss (G) range has been used for treatment of chronic low pack pain disorders [19] we, employed very low amplitude (3.3 × 10^-8^ G to 3.43 × 10^-7^ G) and low-frequency (0.92–7.7 Hz) EMF. The selection of the applied signal was based on other studies that have used similar low-amplitude, low-frequency magnetic fields [20–24]. However, it is still unclear which exact frequencies are of benefit and thus a range of frequencies are used. Nonetheless, on the basis of our findings, it can be suggested that such low-amplitude and low-frequency EMF is safe and may also be effective in the treatment of chronic nonresponsive LBP in the post-treatment period and thus could be further developed as a viable alternative for pain management in disc degenerative disease. It is our contention that both frequency and amplitude are equally important to successful field delivery; the energy delivered at the frequencies used in the present study produced beneficial responses. Since the biological systems under examination are sophisticated and dynamic it is likely that the correctness of a field will vary due to the fluctuating spatiotemporal characteristics of the system. The precise mechanisms of beneficial action remain to be fully elucidated, however; many aspects of cell biology as well as the endogenous control of inflammation, healing and bone remodeling have been reported to be modified in response to EMF [10,25–27]. We believe that the EMF exerts a local effect and thus it is conceivable that there is some reduction in inflammation with some healing resulting in reduced transmission of pain signals to the brain, possibilities that need further examination. Indeed, a better understanding of the mechanisms of beneficial action of EMF could help in identifying patients more likely to benefit from this treatment modality. Furthermore, although humans are continuously exposed to electromagnetic fields, short, controlled exposure to specific electromagnetic fields may exert therapeutic benefits [10].

A limitation of our study is that there were more male subjects in the control group and more female subjects in the EMF group. This imbalance of gender in the groups was a reflection of the randomization process that was undertaken in assigning subjects to the two different groups. While we cannot exclude the possibility that the beneficial effects of EMF in our study may be related to sex, future studies are needed to prove this assumption right or wrong. While the results obtained in the present pilot study are positive in the short term, it is, however; a small study and thus some caution must be exercised in the interpretation of the data obtained. Therefore, a study with a larger patient sample and a long-term (at least 6 months) follow-up period is warranted.

## Conclusion

The pilot data suggest that the EMF treatment protocol used in the present study may have clinical relevance and may emerge as a safe and effective adjuvant option in the approach for management of pain in patients with degenerative disc disease.

**Table 1.** **Electromagnetic field pattern protocol.**

**Magnetic field amplitude (G)**	**Frequency (Hz)**	**Duration (minutes)**
3.3 × 10^-^^8^ G	0.92	15
2.74 × 10^-7^ G	7.7	12
3.43 × 10^-7^ G	9.6	10
3.3 × 10^-8^ G	0.92	23

G: Gauss; Hz: Hertz.

**Table 2.** **Demographic and medical variable comparison of electromagnetic field group and control groups.**

**Variable**	**EMF**	**Control**	**p-value**
Age (years)	60.3 (9.7)	59.1 (13.4)	0.831
Systolic BP (mm Hg)	120.8 (9.9)	122.8 (15.5)	0.929
Diastolic BP (mm Hg)	81.4 (10.0)	75.5 (14.7)	0.216
Heart rate (beats/min)	71.9 (11.3)	77.0 (11.6)	0.387

Differences in the demographic and medical variables at baseline between control and EMF groups were determined by using the independent groups *t*-test for continuous variables. The level of statistical significance was set at p < 0.05.

BP: Blood pressure; EMF: Electromagnetic field therapy.

**Table 3.** **Roland disability questionnaire changes from baseline to end point.**

**Variable**	**Baseline median values (first quartile, third quartile)**	**End point median values (first quartile, third quartile)**	**p-value**
**Total score**			
Control	12.0 (8.0, 19.0)	11.0 (11.0, 17.0)	0.917
Experimental	17.0 (12.0, 21.0)	17.0 (12.0, 20.0)	0.195
**Bothersome index**			
Control	6.0 (2.0, 8.0)	6.0 (1.0, 8.0)	0.657
Experimental	7.0 (2.0, 9.0)	7.0 (2.0, 8.0)	0733

Differences in scores on the Roland Disability Questionnaire between baseline and end point for each group were analyzed by the Wilcoxon matched pairs test on ranks. The level of statistical significance was set at p < 0.05. High scores = poor functioning.

**Table 4.** **SF-36 health survey changes from baseline to end point.**

**Variable**	**Baseline median values (first quartile, third quartile)**	**End point median values (first quartile, third quartile)**	**p-value**
**Control group**			
Physical health total score	47 0 (45.0, 54.0)	49.0 (47.0, 52.0)	0.295
Physical functioning	20.0 (16.0, 23.0)	20.0(15.0, 20.0)	1.000
Role-physical	4.0 (4.0, 5.0)	4.0 (4.0, 5.0)	1.000
Bodily pain	8.0 (7.0, 9.0)	7.0 (7.0, 7.0)	0.173
General health	14.0 (14.0, 19.0)	16.0 (16.0, 22.0)	0.043
Mental health total score	44.0 (42.0, 49.0)	47.0 (46.0, 49.0)	0.447
Vitality	14.0 (13.0, 19.0)	16.0 (15.0, 18.0)	0.590
Social functioning	6.0 (4.0, 6.0)	6.0 (5.0, 7.0)	0.423
Role-emotional	3.0 (3.0, 4.0)	3.0 (3.0, 5.0)	1.000
Mental health	20.0 (18.0, 23.0)	20.0 (18.0, 22.0)	0.686
**EMF group**			
Physical health total score	41.5 (40.0, 45.0)	43.0 (39.0, 46.0)	0. 929
Physical functioning	15.5 12.0, 18.0)	16.5 (15.0, 22.0)	0.022
Role-physical	4.0 (4.0, 4.0)	4.0 (4.0, 5.0)	0.575
Bodily pain	9.0 (8.0, 10.0)	7.5 (6.0, 9.0)	0.045
General health	13.0 (11.0, 14.0)	13.0 (10.0,15.0)	0.906
Mental health total score	45.5 (42.0, 49.0)	46.9 (43.0, 50.0)	0.753
Vitality	15.5 (11.0, 18.0)	14.0 (13.0, 16.0)	0.563
Social functioning	6.5 (6.0, 7.0)	7.0 (6.0, 7.0)	0.799
Role-emotional	4.0 (3.0, 5.0)	4.0 (3.0, 6.0)	0.735
Mental health	20.5 (19, 21)	20.0 (19.0, 23)	0.347

Differences in scores in the SF-36 Health Survey between baseline and end point for each group were analyzed by the Wilcoxon matched pairs test on ranks. The level of statistical significance was set at p <0.05. High scores = poor functioning.

EMF: Electromagnetic field therapy.

**Executive summary**

A double-blind, randomized, placebo-controlled pilot study was conducted to assess the efficacy of electromagnetic field therapy (EMF) for the management of chronic lower back pain associated with some degenerative disc disease.
A larger reduction in the total score was observed in the EMF group.
Overall physical health, social functioning and reduction in bodily pain were observed in the EMF group.
A higher level of pain relief at the target area was observed in the EMF group.
Although a decrease in pain intensity was observed in the EMF group, an increase occurred in the control group.
Left lateral mobility was increased only in the EMF treated group.
EMF treatment may be of benefit to patients with some degenerative disc disease with chronic nonresponsive lower back pain.
